# Prolonged Bedtime Smartphone Use is Associated With Altered Resting-State Functional Connectivity of the Insula in Adult Smartphone Users

**DOI:** 10.3389/fpsyt.2019.00516

**Published:** 2019-07-23

**Authors:** Soo-Hyun Paik, Chang-hyun Park, Jin-Young Kim, Ji-Won Chun, Jung-Seok Choi, Dai-Jin Kim

**Affiliations:** ^1^Department of Psychiatry, Seoul St. Mary’s Hospital, The Catholic University of Korea College of Medicine, Seoul, South Korea; ^2^Department of Psychiatry, SMG-SNU Boramae Medical Center, Seoul, South Korea; ^3^Department of Psychiatry and Behavioral Science, Seoul National University College of Medicine, Seoul, South Korea

**Keywords:** problematic smartphone use, bedtime smartphone use, insula, resting state functional connectivity, fMRI

## Abstract

Prolonged bedtime smartphone use is often associated with poor sleep quality and daytime dysfunction. In addition, the unstructured nature of smartphones may lead to excessive and uncontrolled use, which can be a cardinal feature of problematic smartphone use. This study was designed to investigate functional connectivity of insula, which is implicated in salience processing, interoceptive processing, and cognitive control, in association with prolonged bedtime smartphone use. We examined resting-state functional connectivity (rsFC) of insula in 90 adults who used smartphones by functional magnetic resonance imaging (fMRI). Smartphone time in bed was measured by self-report. Prolonged bedtime smartphone use was associated with higher smartphone addiction proneness scale (SAPS) scores, but not with sleep quality. The strength of the rsFC between the left insula and right putamen, and between the right insula and left superior frontal, middle temporal, fusiform, inferior orbitofrontal gyrus and right superior temporal gyrus was positively correlated with smartphone time in bed. The findings imply that prolonged bedtime smartphone use can be an important behavioral measure of problematic smartphone use and altered insula-centered functional connectivity may be associated with it.

## Introduction

For the last decade, smartphones have become indispensable to our life. According to the South Korean national survey conducted in 2016, 85.0% of those aged over 6 years old possessed their own smartphones, and smartphones have largely replaced personal computer-based online behaviors such as gaming, shopping, banking and searching (1). At the same time, however, the dark sides of smartphone use, including musculoskeletal symptoms and sleep disturbances (2, 3), have been highlighted as well. This phenomenon is often referred as “problematic smartphone use,” which is characterized as excessive and uncontrolled smartphone use that leads to functional impairment (4, 5). Though there is no consensus for defining this phenomenon and much dispute is ongoing to determine whether this is a disease entity or not, it seems necessary to investigate what behavioral manifestations are relevant to represent problematic smartphone use and whether there are any neural correlates that are associated with this behavior.

Bedtime smartphone use has been known to worsen sleep quality and consequent daytime function in adults, and was associated with depressed mood in adolescents (6, 7). Overuse of smartphones in bed was proven to be associated with poor sleep quality, mental health, and delayed chronotype in Japanese college students (8). There underlie several mechanisms for this phenomenon; firstly, the screen light emitted from smartphones significantly suppresses the secretion of melatonin, and consequently disrupts sleep (9). Secondly, the exciting contents obtained from smartphones may induce arousal, fright and stress reactions, which may make it difficult to fall asleep. In addition, bedtime smartphone use is an unstructured leisure activity, which has no predefined starting or ending points, and thus may bring about time displacement (6). Consequently, individuals may be engaged in smartphone use more than they first intended, especially in bed. However, little is known about the meaning of prolonged smartphone use in bed. Thus we focused this behavior and hypothesized that this can be one of the cardinal features of problematic smartphone use. And if so, this behavior may be associated with altered neural correlates.

Resting-state functional connectivity (rsFC) is a potential approach for the assessment of the interaction among brain regions at network levels and is widely used to map the morbid brain (10). Smartphone use necessitates touching the screen, seeing and paying attention to the material, and bedtime smartphone use entails decision-making and cognitive control on whether to continue or stop smartphone use. The insula is known to be an interface of the cognitive, homeostatic and affective systems of the human brain (11), and to integrate external sensory and internal physiological signs in an uncerta*in situ*ation, affecting the decision-making process (12). In addition, the insula has been implicated in salience processing, drug craving, and cognitive control, all of which are fundamental to addictive disorders (13–15). As well, problematic smartphone use seems to have similarities with Internet gaming disorder (IGD) in that both involve online activity and are mediated *via* devices. Zhang and colleagues demonstrated that individuals with IGD exhibited altered insula-centered rsFC compared to healthy controls (16). Thus we considered that insula would be relevant regions of interest. This study was designed to investigate the following two hypotheses: 1) prolonged bedtime smartphone use may serve as one of the relevant behavioral measures of problematic smartphone use, and 2) prolonged bedtime smartphone use may be associated with insula-centered functional connectivity.

## Material and Methods

### Participants

Participants were recruited through an online survey regarding smartphone use. We included all responders who agreed to enroll in this study. We excluded those who failed to fill all the self-report measures and whose head motion artifact was too excessive to be included in the final analysis. From a total of 728 adults who participated in the survey on smartphone usage, 91 smartphone users (33 male and 58 female) were recruited for this functional magnetic resonance imaging (fMRI) study. One female participant was excluded due to excessive head motion during scanning and finally 90 participants were included [the mean age: 26.99, standard deviation (SD): 5.582]. From a total of 90 participants, only one male participant was left-handed.

All study procedures were performed in accordance with the guidelines of the Declaration of Helsinki. The Institutional Review Boards of Seoul St. Mary’s Hospital approved the study protocol (KC15EISI0103). All subjects were informed about the study and all provided informed and written consent and were financially compensated for their time.

### Measures

#### Bedtime Smartphone Use, Sleep Duration and Sleep Latency

Bedtime smartphone use was measured by asking participants how long they used their smartphone in bed before falling asleep, and the answer was manifested as minutes (min).

Sleep duration was measured by asking participants sleep onset and wake up time and calculating the gap between them, weekdays and weekends respectively. Data on sleep latency, a component of the PSQI, were separately collected as well as used to calculate the global sleep quality.

#### Pittsburgh Sleep Quality Index (PSQI)

PSQI, a 19-item scale with seven components (subjective sleep quality, sleep latency, sleep duration, habitual sleep efficiency, sleep disturbances, use of sleep medication, and daytime dysfunction), was used to assess the global sleep quality (17). The sum of each component yields the total score, ranging from 0 to 21, with higher scores indicating poorer global sleep quality. The Korean version of PSQI had good internal consistency (Cronbach’s alpha = 0.84) and reliability and suggested a best cutoff point of 8.5 to distinguish good and poor sleepers (18). Cronbach’s alpha for this study was .599.

#### Smartphone Addiction Proneness Scale (SAPS)

SAPS is a 15-item scale with a four-point Likert scale (1: “not at all” and 4: “always) and widely used for the assessment of smartphone addiction proneness (19). SAPS consists of four subscales, S1 [daytime dysfunction, items 1, 5, 9, 12, and 15 (reverse)], S2 (virtual life orientation, items 2 and 6), S3 [withdrawal, items 4 (reverse), 8, 11, and 14] and S4 [tolerance, items 3, 7, 10 (reverse), and 13] and a higher score indicated higher smartphone addiction proneness. The reliability of the scale revealed a Cronbach’s alpha of .880. Cronbach’s alpha for this study was .943, and Spearman’s correlation coefficients between total and subscale scores were .899, .762, .863, and .880, respectively.

#### Brief Self-Control Scale (BSCS)

BSCS, a 13-item questionnaire with a five-point Likert scale (1: “strongly disagree” and 5: “strongly agree”), was used to measure self-control ability (20). BSCS measures the ability to override or change one’s inner response as well as to interrupt undesired behavioral tendencies and refrain from acting on them, with higher scores indicating lower self-control ability. BSCS had good internal consistency (Cronbach’s alpha = 0.85) in the original study. Cronbach’s alpha was .880 in this study.

#### Imaging Data Acquisition

Resting-state fMRI (rsfMRI) data were acquired using a 3T Siemens MRI system (Siemens, MAGNETOM Verio, Erlangen, Germany) equipped with a 16-channel head coil. Participants’ heads were cushioned with attached earmuffs. Participants were instructed to keep their head still and eyes open during scanning. The functional images were obtained using a T2*-weighted gradient echo-planar imaging sequences [37 slices, slice thickness = 4.0mm, no gaps, repetition time (TR) = 2,000 ms, echo time (TE) = 30 ms, flip angle = 90°, field of view (FOV) = 240 mm, acquisition matrix = 64 × 64, voxel size = 3.8 × 3.8 × 4.0 mm^3^, time point = 200]. A high-resolution T1-weighted image was obtained to permit functional localization (192 slices, slice thickness = 1.0 mm, TR = 2,300 ms, TE = 2.52 ms, flip angle = 9°, FOV = 256 mm, voxel size = 1.0 × 1.0 × 1.0 mm^3^).

#### Imaging Data Preprocessing and Analysis

Imaging data were preprocessed and analyzed using Statistical Parametric Mapping software (SPM12; http://www.fil.ion.ucl.ac.uk/spm/; Wellcome Trust Centre for Neuroimaging, University College London, London, UK) and DPABI version 3.0 (http://rfmri.org/dpabi). After discarding the first five time points, differences in image acquisition time between slices were corrected. Realignment was performed to minimize head motion artifact and the corrected images were coregistered onto the T1-weighted image of each participant. The T1-weighted images were normalized to the Montreal Neurological Institute (MNI) space and the resulting transformation matrices were applied to the coregistered functional images. rsFMRI data were smoothed with a Gaussian kernel of 6 mm full-width at half maximum, bandpass filtered (0.009–0.08 Hz), and linearly detrended. Signals from rigid body 6 motions, white matter, cerebrospinal fluid and global motion were removed *via* nuisance regression.

To compute the rsFC of the insula, regions of interest (ROIs) were defined by anatomically-defined insula using the Automated Anatomical Labeling (AAL) atlas. The average time series within each seed was correlated with time-series of every voxel in the whole brain to generate cross correlation maps. Voxel-wise correlation coefficients were converted to *Z*-score *via* Fisher’s *r*-to-*Z* transformation.

Whole brain voxel-wise regression analyses were performed to examine whether there was a correlation between rsFC and bedtime smartphone use with age and gender as nuisance covariates with a family-wise error (FWE) corrected cluster-level threshold of *p* < 0.05 determined at a height threshold of uncorrected *p* < 0.001. Pearson correlation analyses between SAPS, smartphone time (weekday and weekend), smartphone time in bed, PSQI, sleep latency, sleep duration (weekday and weekend), and BSCS were performed. Correlation analyses were performed between parameters extracted from multiple regression analyses (FC *z*-scores) and SAPS, total smartphone time, PSQI and BSCS. FDR multiple comparison corrections were conducted to the value of correlation using the Benjamini–Hochberg procedure.

Demographic and clinical data of the sample were analyzed quantitatively and qualitatively using R Statistical Software (Foundation for Statistical Computing, Vienna, Austria).

## Results

### Sample Characteristics

Demographic characteristics of 90 adult smartphone users and the correlation between variables were shown in **Table 1**. The mean SAPS score was 36.92 (SD = 11.354) and scores for subscales were 11.61 ± 4.016 for S1 (daytime dysfunction), 3.99 ± 1.673 for S2 (virtual life orientation), 10.18 ± 3.74 for S3 (withdrawal) and 11.14 ± 3.59 for S4 (tolerance). Weekday and weekend smartphone time were 4.14 ± 2.48 hours (h) and 4.52 ± 2.67 h respectively. Participants spent 62.3 ± 53.43 minutes (min) using their smartphone in bed. The median value for smartphone use in bed was 60.00 min. The mean sleep latency was 30.01 ± 24.39 min. PSQI and BSCS total scores were 6.70 ± 3.14 and 37.87 ± 8.219 respectively. Among all participants, 32 (35.6%) participants never delayed sleep due to smartphone use while 58 (64.4%) delayed at least once or more in a week. Specifically, 37 (41.1%) participants delayed 1–2 times per week, 17 (18.9%) delayed 3-4 times per week, and 4 (4.4%) delayed 5–7 times per week. When we analyzed SAPS and smartphone time according to this classification, those who delayed sleep due to smartphone use 5–7 times per week had the highest SAPS score (Never: 31.50 ± 11.254, 1–2 times/week: 37.35 ± 10.541, 3–4 times/week: 41.76 ± 6.915, and 5–7 times/week: 55.75 ± 2.363, *F* = 9.100, *p* < .005, *post hoc* Bonferroni: Never < 3–4 times/week, 1–2 times/week < 5–7 times/week) and smartphone time in bed (Never: 44.75 ± 42.78 min, 1–2 times/week: 60.41 ± 57.71 min, 3–4 times/week: 84.12 ± 43.88 min, and 5–7 times/week: 127.50 ± 61.85 min, F = 4.594, p < .05, *post hoc* Bonferroni: Never < 5–7 times/week), but total smartphone time did not differ across groups.

**Table 1:** Sample characteristics.

Variables	Overall
**Age**	26.99 ± 5.582
**Male (%)**	33 (36.7%)
**SAPS**	36.92 ± 11.354
** S1 (daytime dysfunction)**	11.61 ± 4.016
** S2 (virtual life orientation)**	3.99 ± 1.673
** S3 (withdrawal)**	10.18 ± 3.74
** S4 (tolerance)**	11.14 ± 3.59
**Weekday smartphone time (hours)**	4.14 ± 2.48
**Weekend smartphone time (hours)**	4.52 ± 2.67
**Weekday sleep duration (hours)**	7.112 ± 1.248
**Weekend sleep duration (hours)**	8.142 ± 1.109
**Smartphone time in bed (minutes)**	62.3 ± 53.43
**PSQI total score**	6.70 ± 3.14
** C1 (subjective sleep quality)**	1.33 ± 0.62
** C2 (sleep latency)**	1.41 ± 0.95
** C3 (sleep duration)**	1.19 ± 1.19
** C4 (habitual sleep efficiency)**	0.67 ± 1.11
** C5 (step disturbance)**	1.18 ± 0.44
** C6 (use of sleeping medication)**	0.05 ± 0.27
** C7 (daytime dysfunction)**	0.87 ± 0.77
**Sleep latency (minutes)**	30.01 ± 24.39
**BSCS**	37.87 ± 8.219
**Delayed sleep due to smartphone use**	
** Never**	32 (35.6%)
** 1–2 times/week**	37 (41.1%)
** 3–4 times/week**	17 (18.9%)
** 5–7 times/week**	4 (4.4%)

SAPS, Smartphone Addiction Proneness Scale; PSQI, Pittsburgh Sleep Quality Index; BSCS, Brief Self-Control Scale.

**Table 2** showed the correlation between variables. Smartphone time in bed was positively correlated with SAPS and total smartphone time, but not with PSQI total score, sleep duration and latency, and BSCS.

**Table 2:** Correlation between variables.

	1	2	3	4	5	6	7	8	9
**1. SAPS**	1								
**2. Weekday smartphone time**	.383*	1							
**3. Weekend smartphone time**	.389**	.876**	1						
**4. Smartphone time in bed**	.395**	.305*	.354**	1					
**5. PSQI**	.350**	.154	.068	.118	1				
**6. sleep latency**	.199	.229	.255	−.099	.289*	1			
**7. Weekday sleep duration**	−.204	−.158	−.191	−.103	−.193	−.058	1		
**8. Weekend sleep duration**	-.005	.016	.000	.000	−.172	.102	.387**	1	
**9. BSCS**	.554**	.143	.136	.139	.454**	.210	.037	.043	1

SAPS, Smartphone Addiction Proneness Scale; PSQI, Pittsburgh Sleep Quality Index; BSCS, Brief Self-Control Scale.

**p < .005, *p < .05.

### rsFC Results

**Figure 1:**
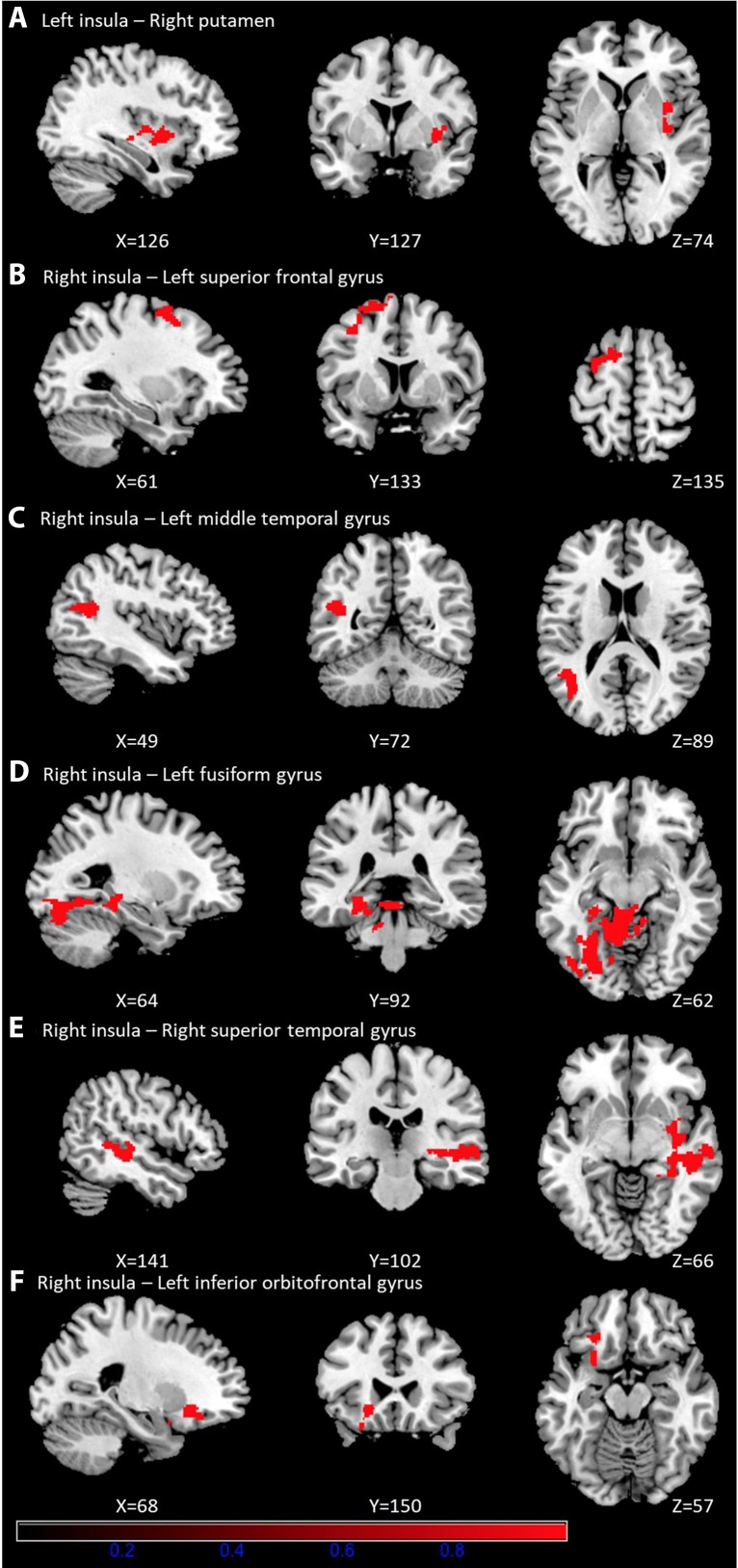
Brain regions showing significantly positive correlation with smartphone time in bed. Prolonged bedtime smartphone use was associated with increased functional connectivity of the left insula with the right putamen **(A)**, and of the right insula with the left superior frontal gyrus **(B)**, left middle temporal gyrus **(C)**, left fusiform gyrus **(D)**, right superior temporal gyrus **(E)**, and left inferior orbitofrontal gyrus **(F)**.

As shown in **Table 3**, prolonged smartphone time in bed was positively correlated with insula-centered rsFC. Prolonged bedtime smartphone use was associated with increased functional connectivity of the left insula with the right putamen, and of the right insula with the left superior frontal gyrus, left middle temporal gyrus, left fusiform gyrus, right superior temporal gyrus, and left inferior orbitofrontal gyrus. (**Figure 1**).

**Table 3:** Seed locations and regions showing significantly positive correlation with smartphone time in bed.

Seed	Region	Brodmann area	Cluster size	Peak MNI (mm)			Peak *T*- score
				*x*	*y*	*z*	
L insula	R putamen	49	195	36	4	−2	4.00
R insula	L superior frontal	4	773	−44	−14	50	4.44
	L middle temporal	39	237	−44	−54	18	4.43
	L fusiform	18	1632	−24	−76	−12	4.38
	R superior temporal	21	1064	44	−32	−2	4.35
	L inferior orbitofrontal	47	194	−20	24	-8	4.18

L, left; R, right.

### Relationship Between rsFC Strength and Clinical Characteristics

**Figure 2:**
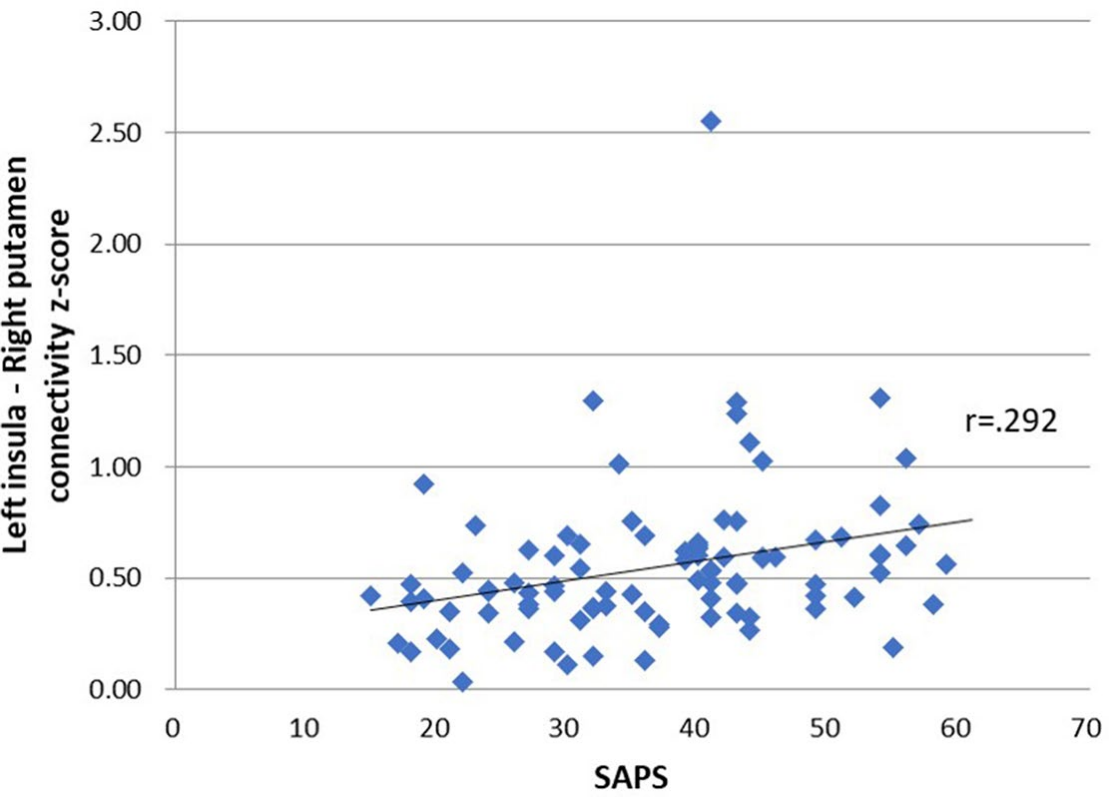
Relationship between seed-region of interest (ROI) functional connectivity and smartphone addiction proneness scale (SAPS) scores. SAPS scores were positively correlated with connectivity *z*-score of left insula and right putamen, *r* = .292, *p* = .03.

**Table 4** shows the relationship between seed-ROI rsFC and SAPS, weekday and weekend smartphone time, PSQI and BSCS. Smartphone addiction proneness was positively correlated with rsFC between the left insula and right putamen (**Figure 2**). Total smartphone time, PSQI and BSCS were not correlated with the strength of insula-ROI rsFC.

**Table 4:** Relationship between seed-ROI functional connectivity and other variables.

Seed	ROI	SAPS	Weekday smartphone time	Weekend smartphone time	PSQI	BSCS
**L insula**	**R putamen**	.292*	−.010	.019	.106	.152
**R insula**	**L fusiform**	.145	−.025	−.001	.027	.048
	**L inferior orbitofrontal**	.153	.006	.019	.037	.069
	**L middle temporal**	.161	−.051	−.063	.113	.090
	**L superior frontal**	.229	−.005	.054	−.025	.058
	**R superior temporal**	.172	−.016	.006	.027	.028

ROI, region of interest; SAPS, Smartphone Addiction Proneness Scale; PSQI, Pittsburgh Sleep Quality Index; BSCS, Brief Self-Control Scale; L, left; R, right. **p < .005, *p < .05.

## Discussion

In this study, we found that smartphone use in bed was positively correlated with smartphone addiction proneness and was associated with enhanced functional connectivity of the insula with a network of brain regions. Specifically, the rsFC between the left insula and the right putamen, and the right insula and the left superior frontal, middle temporal, fusiform and orbital frontal gyrus and right superior temporal gyrus was enhanced. In addition, the strength of rsFC between the left insula and the right putamen, and the right insula and the left superior frontal gyrus was positively correlated with smartphone addiction proneness.

Smartphone time in bed was positively correlated with smartphone addiction proneness scale and its subscales, and total smartphone time. In contrast to previous studies, bedtime smartphone use was not correlated with general sleep quality, duration, and latency (6, 7). This discrepancy may be due to the difference in sample characteristics. While previous studies focused on adolescents or adults with a broad age range of 18 to 94 years, our study included those aged from 20 to 39 years old. While boy adolescents are well known for the dramatic shift to eveningness and older people are prone to morningness (21, 22), adults in our sample age mostly have an intermediate chronotype. We also found that individuals who postponed their sleep due to smartphone use most frequently showed the highest smartphone addiction proneness score and smartphone time in bed, but total smartphone time did not differ. Though these results need further validation because of the small numbers allocated in each group, this poses an insight that individuals with longer duration of bedtime smartphone use may feel more difficulty to cut down their smartphone use in bed. Taken together, the results suggested that prolonged bedtime smartphone use may be associated with problematic smartphone use, without influencing sleep quality, and may serve as one of the relevant behavioral measures of problematic smartphone use.

Longer bedtime smartphone use was associated with greater strength in five insula-centered rsFC. First, the rsFC between the left insula and right putamen was enhanced in relation to prolonged smartphone use in bed. The striatum is a brain region that dominates the reward processing and both the insula and putamen are involved in time perception (23, 24). Decreased insula-striatum connectivity has been observed in IGD (25, 26), a direction opposite to this study. While stressful situations are associated with the experience of time passing more slowly, relaxed feeling states are associated with experience of passing time more rapidly (27). These findings pose a possibility that feeling relaxed in bed may be associated with longer smartphone time due to greater perceived quickness, and time spent more than they first intended may be associated with increased rsFC between the left insula and right putamen. Second, rsFC of the right insula with left superior frontal gyrus (SFG) and inferior orbitofrontal gyrus (OFG) was increased. The insula is implicated in higher order cognitive functions with a network of brain regions; in association with insula, and IFG is in decision-making and evaluation of risk (13, 28), particularly subjective risk prediction which is influenced by pessimism/optimism or risk aversion/tolerance (28). SFG is involved in planning, motivation, and emotional information processing and the left SFG is particularly involved in spatially orienting processes (29, 30). These findings imply that prolonged bedtime smartphone use would be associated with altered higher order cognitive function. Third, rsFC between the right insula and right superior temporal gyrus (STG) and left middle temporal and fusiform gyrus and right superior temporal gyrus (STG) was increased. A resting brain study on human insula showed that the salience network is lateralized to the right and displays strong connectivity with the left frontal cortex, while the visuomotor integration network has stronger connection with superior temporal cortex and occipital cortex on the right (31). The insula is the brain region in which visceral sensation from temporal cortex and emotional components from the amygdala, nucleus accumbens, orbitofrontal cortex is conveyed to and integrated. While STG serves to process audiovisual information (32), the middle temporal and fusiform gyrus are involved in visual processing, including color information processing, face and body recognition, and word recognition (33, 34). In addition, both STG and insula are involved in the decision-making process, particularly in switching responses relative to staying with the same choice (35). Taken together, these findings support that prolonged bedtime smartphone use would be associated with enhanced visceral sensation processing and cognitive overloading, particularly in complex decision making. These findings were also consistent with results observed in IGD (16).

Interestingly, the rsFC between the left insula and right putamen were positively correlated with smartphone addiction proneness. Morbid brains sometimes have enhanced functional connectivity between specific brain regions, which may seem counterintuitive. Since morbid brains may need more cognitive efforts due to inefficiency, enhanced connectivity may support this cognitive overloading, as is the case of IGD and obsessive compulsive disorder (16, 36). Thus, the alteration of insular connectivity in association with prolonged bedtime smartphone use may serve as an indicator of the severity of problematic smartphone use.

As we hypothesized, the functional connectivity of the insula was associated with prolonged smartphone use in bed. The insula has been implicated in the cognitive, affective, and regulatory functions and provides a link between stimulus-driven processing and brain regions involved in monitoring the internal milieu (11). Insula-mediated interoceptive representations have the capacity to “hijack” the cognitive resources necessary for exerting inhibitory control to resist the temptation to smoke, use drugs, or use social media impulsively by disabling the activity of the prefrontal system (37). In IGD, the insula seems to play a crucial role *via* the interoceptive system in modulating equilibrium between impulsive and reflective systems (38). Smartphone use in bed requires integrating external stimuli obtained from smartphones with internal milieu such as balancing between emotional or physical excitement and the need for sleep in appropriate timing. Individuals with problematic smartphone use may necessitate more cognitive effort to manage integrative and interoceptive function than those without.

Several limitations should be noted. First, the cross-sectional nature of this study could not confirm the causal relationship between altered insula-centered rsFC and prolonged smartphone use in bed. Second, we did not screen psychiatric comorbidity including substance use disorder. Third, the amount of smartphone use relied upon self-reporting, and there was no information on smartphone contents that were consumed in bed. Fourth, the amount of smartphone use in bed may also be a part of total daytime smartphone use. However, when classified by the frequency of delaying sleep due to smartphone use, smartphone time in bed was higher in those who delayed sleep most frequently while total smartphone time did not differ. This implies that total smartphone time and smartphone time in bed may have different function on problematic smartphone use. Fifth, we did not have comparative healthy control subjects since the sample was not recruited through clinics, and there is much dispute on defining problematic smartphone use. Thus, our findings are confined to the correlational interpretation. Lastly, functional connectivity is correlational and does not determine the direction between nodes. Further research using effective connectivity would validate the altered direction of the functional connectivity between nodes manifested in individuals with prolonged bedtime smartphone use.

Despite the shortcomings, this study had intriguing strengths. First, to the best of our knowledge, this is the first study investigating the neural correlates of prolonged bedtime smartphone use among adult smartphone users. Second, we explored the alteration in resting-state functional connectivity in association with one particular behavior. Despite increased attention on problematic smartphone use and devoted efforts to define this phenomenon, there is still no standardized consensus for problematic smartphone use. By investigating the neural substrates relevant to prolonged bedtime smartphone use, we deliberately suggested altered insular functional connectivity may be associated with problematic smartphone use.

## Ethics Statement

All study procedures were performed in accordance with the guidelines of the Declaration of Helsinki. The Institutional Review Boards of Seoul St. Mary’s Hospital approved the study protocol (KC15EISI0103). All subjects were informed about the study and all provided informed consent and were financially compensated for their time.

## Author Contributions

S-HP, C-HP, J-SC and D-JK contributed to the conception and design of this study. J-YK and J-WC contributed to the acquisition of behavioral and imaging data. S-HP performed clinical and imaging data analysis. S-HP wrote the first version of this manuscript and prepared the figures and tables. C-HP, J-YK and J-WC assisted with the interpretation of the data. All authors contributed to the revision of the manuscript critically and approved the final manuscript.

## Funding

This research was supported by the Brain Research Program through the National Research Foundation of Korea (NRF) funded by the Ministry of Science, ICT & Future Planning (NRF-2014M3C7A1062893).

## Conflict of Interest Statement

The authors declare that the research was conducted in the absence of any commercial or financial relationships that could be construed as a potential conflict of interest.
